# The Impact of the COVID-19 Pandemic on Ambulatory Lumbar Spine Decompression Surgery

**DOI:** 10.7759/cureus.60058

**Published:** 2024-05-10

**Authors:** Yusuke Dodo, Ichiro Okano, Neil A Kelly, Leonardo A Sanchez, Henryk Haffer, Maximilian Muellner, Erika Chiapparelli, Lisa Oezel, Gisberto Evangelisti, Jennifer Shue, Darren R Lebl, Frank P Cammisa, Federico P Girardi, Alexander P Hughes, Gbolabo Sokunbi, Andrew A Sama

**Affiliations:** 1 Spine Care Institute, Hospital for Special Surgery, New York, USA; 2 Department of Orthopaedic Surgery, Showa University, Tokyo, JPN

**Keywords:** trends, length of stay, pandemic, covid-19, ambulatory spine surgery

## Abstract

Background

Only a few studies have examined the impact of the coronavirus disease 2019 pandemic on spine ambulatory surgeries and changes in trends. Therefore, we investigated trends during the pre-pandemic period and three pandemic stages in patients undergoing lumbar decompression procedures in the ambulatory surgery (AMS) setting.

Methodology

A total of 2,670 adult patients undergoing one- or two-level lumbar decompression surgery were retrospectively reviewed. Patients were categorized into the following four groups: 1: pre-pandemic (before the pandemic from January 1, 2019, to March 16, 2020); 2: restricted period (when elective surgery was canceled from March 17, 2020, to June 30, 2020); 3: post-restricted 2020 (July 1, 2020, to December 31, 2020, before vaccination); and 4: post-restricted 2021 (January 1, 2021 to December 31, 2021 after vaccination). Simple and multivariable logistic regression analyses as well as retrospective interrupted time series (ITS) analysis were conducted comparing AMS patients in the four periods.

Results

Patients from the restricted pandemic period were younger and healthier, which led to a shorter length of stay (LOS). The ITS analysis demonstrated a significant drop in mean LOS at the beginning of the restricted period and recovered to the pre-pandemic levels in one year. Multivariable logistic regression analyses indicated that the pandemic was an independent factor influencing the LOS in post-restricted phases.

Conclusions

As the post-restricted 2020 period itself might be independently influenced by the pandemic, these results should be taken into account when interpreting the LOS of the patients undergoing ambulatory spine surgery in post-restricted phases.

## Introduction

The coronavirus disease 2019 (COVID-19) outbreak spread rapidly worldwide and impacted human life on an unprecedented scale since the first reported case to the World Health Organization (WHO) in December 2019 [1]. COVID-19 disrupted millions of people’s day-to-day lives, businesses, and healthcare services [2]. During the pandemic, elective surgical procedures were interrupted in hospitals and ambulatory surgery (AMS) centers. In an attempt to maximize medical resources while minimizing the transmission of the virus, elective scheduled surgeries were postponed or canceled [3,4].

At the beginning of the pandemic, the Centers for Medicare and Medicaid Services (CMS) and the Surgeon General’s office announced a plan for all elective and non-essential surgeries and procedures to be delayed to enhance the response to COVID-19 [5]. During the pandemic, the CMS classified surgeries into the following three main tiers: (1) Tier 1 recommended to postpone surgery, (2) Tier 2 recommended to consider postponing surgery, and (3) Tier 3 recommended not to postpone surgery. In the three-tiered CMS hierarchy, spine surgery was included in Tier 2, along with a recommendation to consider postponing surgery for an illness that is not life-threatening but has the potential for future morbidity and mortality [5]. These CMS recommendations for the practical management and triaging of surgical indications presented clinical and ethical challenges for spine surgeons [5]. Generally, early surgical intervention is necessary for patients with neurological deficits to improve clinical and neurological outcomes and reduce overall healthcare costs [6]. However, the pandemic changed the indication criteria for spine surgery [5,7,8].

A recent study of safe spine surgery management during the COVID-19 pandemic recommended that certain essential (emergency and urgent) procedures be performed to prevent permanent long-term disability or death for patients even during the pandemic [9]. Despite medical teams in full personal protective equipment, hospitals were still considered high-risk locations for COVID-19 transmission. The safest place during the pandemic was thought to be an individual’s own home.

During the pandemic, there has been an abundance of literature published on COVID-19 and AMS. However, only a few studies have examined the impact of the COVID-19 pandemic on spine AMSs and changes in trends. Therefore, this study aimed to investigate the impact of the pandemic on trends and characteristics of patients undergoing one- or two-level lumbar decompression procedures intended for AMS.

This article was previously presented as a poster presentation at the 2022 International Society for the Advancement of Spine Surgery Annual Meeting on June 1-4, 2022, and at the 2023 Global Spine Congress Annual Meeting on May 31-June 3, 2023.

## Materials and methods

This is a single-center, retrospective, observational study. The investigation followed the Declaration of Helsinki and was approved by our hospital’s institutional review board. Informed consent of each patient was waived given the retrospective nature of this study. The electronic medical records of all patients who underwent ambulatory lumbar decompression surgery between January 1, 2019, and December 31, 2021, were reviewed. The type of surgery included micro-laminectomy, microdiscectomy, laminectomy, discectomy, foraminotomy, cyst excision, and exploration for post-operation hematoma. Exclusion criteria included (1) operated disc level of more than two levels, (2) any types of fusion surgery, (3) revision surgery, (4) non-decompression procedure, such as kyphoplasty or implant removal, and (5) patients under 18 years old.

In line with the CMS announcement, elective surgeries were considered restricted from March 17, 2020, to June 30, 2020. The restriction was lifted in July 2021 and all elective surgeries were managed and scheduled as they were before the pandemic [5]. The first COVID-19 vaccination was approved for emergency use at the end of December 2020 [10]. Patients were categorized into four phases based on the period of the pandemic as follows: (1) pre-pandemic period (January 1, 2019, to March 16, 2020), (2) restricted period (March 17, 2020, to June 1, 2020), (3) post-restricted period 2020 (July 1, 2020, to December 31, 2020) defined as after the restricted period but before the vaccination period, and (4) post-restricted period 2021 (January 1, 2021, to December 31, 2021) defined as after the vaccination period. Patients were assigned to one of these four categories based on the date of the surgical procedure.

Surgery case numbers for the pre-pandemic, restricted, post-restricted 2020, and post-restricted 2021 period from 2019 through 2021 were collected. During the restricted period, our institution followed the CMS recommendation for spine surgery indications. Hence, the restricted period was further classified into the following three phases: “Emergent” from March 17, 2020, to May 10, 2020, “Urgent” from May 11, 2020, to June 1, 2020, and “Urgent-Elective” from June 1, 2020, to June 28, 2020. For patient classification based on admission time, patients discharged within 24 hours after surgery were defined as AMS. Patients who required an extended hospital stay for additional medical care of more than one night but less than a 48-hour stay after surgery were defined as observation service (OS). Patients staying greater than 48 hours were defined as inpatients.

Data collection

Data on patient demographics, medical comorbidities, length of stay (LOS) (hours), admission patient class, surgical information, and administrative information were retrospectively collected. Demographic variables included LOS, admission class, age, gender, body mass index (BMI), race, the American Society of Anesthesiologists Physical Status (ASA) classification, insurance status, marital status, smoking status, opioid use (oxycodone 4 mg per day or more), and steroid use. The ASA score indicates physiological status and is often used for preoperative risk management [11]. Medical comorbidities included the Charlson Comorbidity Index (CCI) score, which is indicative of long-term mortality and often used for treatment before invasive surgery [12]. The data for other comorbidities including coronary artery disease, gastroesophageal reflux disease, COVID-19, human immunodeficiency virus, hyperlipidemia, obesity, osteoporosis, Parkinson’s disease, rheumatoid arthritis, obstructive sleep apnea syndrome, anxiety, depression, arthritis, asthma, autoimmune disease, decreased hearing loss, decreased vision loss, chronic pain, hypertension, and hypothyroidism were collected. Surgery-related factors included drain use, dural tear, procedure type, estimated blood loss (EBL), procedure time (minutes), number of operated levels (one or two levels), operated levels (L1-2, L2-3, L3-4, L4-5, L5-S1), and operation start and end time classified into the following four categories based on staffing shifts: (1) morning before 12:00 p.m., (2) early afternoon from 12:00 p.m. to 3:30 p.m., (3) late afternoon from 3:30 p.m. to 5:30 p.m., and (4) night shift after 5:30 p.m.

Statistical methods

Based on the normality of distribution, mean and standard deviation (SD) or median and range were used for the description of continuous variables. For missing data points, categories indicating missing data were created and separately analyzed as categorical variables. For continuous variables, missing data points were observed only in a few data points (<1%) in one variable and imputation with mean value was performed. Comparisons between continuous variables were performed using the Student’s t-test, analysis of variance, the Mann-Whitney U-test, or the Kruskal-Wallis test based on the distribution and number of comparisons. Simple and multivariable logistic regression analysis as well as interrupted time series (ITS) analysis were conducted comparing AMS patients in pre-pandemic, restricted, and post-restricted stages. Variables for multivariable analyses were chosen. All statistical analyses were performed using R software (R for 4.1.2, r-project.org). Statistical significance was defined as p-values <0.05.

## Results

**Figure 1:**
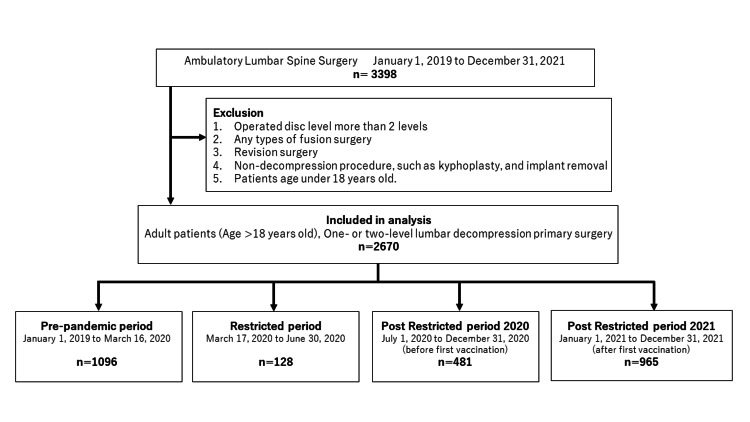
Flow of cases through selection for analysis

Of 3,398 ambulatory lumbar surgery patients, 2,670 adult patients met the inclusion criteria (Figure 1). The 2,670 patients were categorized into one of four time periods. In total, 1,096 patients were classified as pre-pandemic, 128 as restricted period, 481 as post-restricted 2020, and 965 as post-restricted 2021. The number of types of surgeries included micro-laminectomy at 223 cases, microdiscectomy at 1,414 cases, laminectomy at 396 cases, discectomy at 601 cases, foraminotomy at 14 cases, cyst excision at 19 cases, and exploration for post-operation wound at three cases. Overall, 67.9% of the cases (87 out of 128 cases) were microdiscectomy and micro-laminectomy in the pandemic period.

Demographic data is shown in Table 1. A significant difference in the LOS, admission class, age, insurance, marital status, and opioid use was found. The restricted, post-restricted 2020, and post-restricted 2021 periods had a lower admission rate ranging from 7.8% to 9.1% compared to the pre-pandemic period (14.1%). Patients were younger and the LOS was shorter during the restricted period.

**Table 1:** Patient demographics. *: Bold values indicate significance (p < 0.05) LOS: length of stay, AMS: ambulatory surgery (discharge within 24 hours), OS: observation stay (discharge from 24 hours to 48 hours), inpatient: discharged after 48 hours. BMI = body mass index; ASA = American Society of Anesthesiologist physical status; WC = workers compensate; LOS = length of stay

Factor	Group	Pre-pandemic	Restricted Period	Post-restricted 2020	Post-restricted 2021	P-value*
Patients (N)		1,096	128	481	965	
LOS (hours)	Median (range)	25.45 (5.85, 245.27)	10.80 (5.87, 318.00)	16.62 (5.93, 216.00)	24.17 (2.32, 460.13)	<0.001
Admission class, n (%)	AMS	455 (41.5)	81 (63.3)	234 (48.6)	434 (45.0)	<0.001
	OS	486 (44.3)	37 (28.9)	203 (42.2)	444 (46.0)	
	Inpatient	155 (14.1)	10 (7.8)	44 (9.1)	87 (9.0)	
Age	Median (range)	56.00 (18.00, 94.00)	52.00 (18.00, 87.00)	58.00 (18.00, 87.00)	58.00 (18.00, 91.00)	0.001
Gender, n (%)	Male	678 (61.9)	86 (67.2)	291 (60.5)	620 (64.2)	0.165
	Female	410 (37.4)	41 (32.0)	189 (39.3)	344 (35.6)	
	Unspecified	8 (0.7)	1 (0.8)	1 (0.2)	1 (0.1)	
BMI, n (%)	Median (range)	27.10 (16.00, 53.20)	27.00 (19.00, 45.30)	27.00 (17.50, 53.60)	27.20 (15.30, 56.20)	0.724
	Underweight (BMI: -18.4)	12 (1.1)	0 (0.0)	7 (1.5)	10 (1.0)	0.195
	Normal (BMI: 18.5–24.9)	322 (29.4)	39 (30.5)	138 (28.7)	305 (31.6)	
	Overweight (BMI: 25.0–29.9)	476 (43.4)	56 (43.8)	191 (39.7)	352 (36.5)	
	Obesity (BMI: 30–39.9)	267 (24.4)	31 (24.2)	135 (28.1)	272 (28.2)	
	Morbid obesity (BMI: 40)	19 (1.7)	2 (1.6)	10 (2.1)	26 (2.7)	
Race, n (%)	White	920 (83.9)	110 (85.9)	407 (84.6)	805 (83.4)	0.385
	Black	42 (3.8)	5 (3.9)	24 (5.0)	32 (3.3)	
	Asian	46 (4.2)	5 (3.9)	16 (3.3)	30 (3.1)	
	Others	88 (8.0)	8 (6.2)	34 (7.1)	98 (10.2)	
ASA, n (%)	1	178 (16.2)	25 (19.5)	68 (14.1)	129 (13.4)	0.062
	2	852 (77.7)	98 (76.6)	373 (77.5)	754 (78.1)	
	3 or over	66 (6.0)	5 (3.9)	40 (8.3)	82 (8.5)	
Insurance, n (%)	Medicare	233 (21.3)	14 (10.9)	112 (23.3)	228 (23.6)	0.007
	Medicaid	1 (0.1)	1 (0.8)	0 (0.0)	1 (0.1)	
	Private	813 (74.2)	107 (83.6)	347 (72.1)	685 (71.0)	
	WC	11 (1.0)	2 (1.6)	13 (2.7)	11 (1.1)	
	Others	10 (0.9)	0 (0.0)	1 (0.2)	11 (1.1)	
	Several	28 (2.6)	4 (3.1)	8 (1.7)	29 (3.0)	
Marital status, n (%)	Single	241 (22.0)	30 (23.4)	100 (20.8)	163 (16.9)	<0.001
	Married	720 (65.7)	91 (71.1)	318 (66.1)	669 (69.3)	
	Divorced	56 (5.1)	1 (0.8)	22 (4.6)	50 (5.2)	
	Widowed	38 (3.5)	3 (2.3)	20 (4.2)	26 (2.7)	
	Unspecified	23 (2.1)	0 (0.0)	6 (1.2)	10 (1.0)	
	Others	18 (1.6)	3 (2.3)	15 (3.1)	47 (4.9)	
Smoking status, n (%)	Never smoker	699 (63.8)	82 (64.1)	302 (62.8)	645 (66.8)	0.169
	Current smoker	74 (6.8)	6 (4.7)	23 (4.8)	44 (4.6)	
	Former smoker	289 (26.4)	35 (27.3)	146 (30.4)	255 (26.4)	
	Heavy smoker	13 (1.2)	4 (3.1)	4 (0.8)	8 (0.8)	
	Light smoker	21 (1.9)	1 (0.8)	6 (1.2)	13 (1.3)	
Opioid use, n (%)		319 (29.2)	25 (20.3)	145 (30.4)	347 (36.1)	<0.001
Steroid use, n (%)		67 (6.2)	8 (7.0)	17 (3.7)	71 (7.5)	0.052

Table 2 shows patient comorbidities. There were significant differences among the patient groups in CCI score, age, history of myocardial infarction, congestive heart failure, peripheral vascular disease, COVID-19, asthma, and decreased vision loss. The restricted period showed a significantly lower CCI score.

**Table 2:** Patient comorbidities. *: Bold values indicate significance (p < 0.05). AMS = Ambulatory status (discharge within 24 hours), OS: observation stay (discharge from 24 hours to 48 hours), inpatient: discharged after 48 hours. CCI = Charlson Comorbidity Index; MI = myocardial infarction; CHF = congestive heart failure; PVD = peripheral vascular disease; CVD = cerebrovascular disease; COPD = chronic obstructive pulmonary disease; DM = diabetes mellitus; CKD = chronic kidney disease; AIDS = acquired immunodeficiency syndrome; CAD = coronary artery disease; GERD = gastroesophageal reflux disease; HIV = human immunodeficiency virus; HL = hyperlipidemia; RA = rheumatoid arthritis; OSAS = obstructive sleep apnea

Factor	Group	Pre-pandemic	Restricted period	Post-restricted 2020	Post-restricted 2021	P-value*
Patients (N)		1,096	128	481	965	
CCI score, n (%)	Median (range)	1.00 (0.00, 9.00)	1.00 (0.00, 7.00)	2.00 (0.00, 8.00)	2.00 (0.00, 10.00)	0.002
	Score 0	351 (32.0)	51 (39.8)	133 (27.7)	249 (25.8)	0.008
	Score 1	201 (18.3)	22 (17.2)	89 (18.5)	190 (19.7)	
	2 or over	544 (49.6)	55 (43.0)	259 (53.8)	526 (54.5)	
MI, n (%)		14 (1.4)	1 (1.6)	15 (3.3)	13 (1.5)	0.039
CHF, n (%)		3 (0.4)	2 (2.3)	3 (0.8)	1 (0.2)	0.007
PVD, n (%)		1 (0.0)	0 (0.8)	6 (1.5)	2 (0.3)	<0.001
Cerebrovascular disease, n (%)		8 (0.8)	3 (3.1)	5 (1.2)	7 (0.8)	0.082
Dementia, n (%)		0 (0.1)	0 (0.0)	0 (0.0)	0 (0.0)	0.697
COPD, n (%)		6 (0.6)	0 (0.8)	6 (1.5)	9 (1.0)	0.461
Connective tissue disease, n (%)		20 (1.9)	1 (0.0)	9 (2.1)	12 (1.3)	0.293
Peptic ulcer disease, n (%)		30 (2.8)	0 (0.8)	10 (2.3)	20 (2.2)	0.471
Mild liver disease, n (%)		19 (1.8)	0 (0.8)	7 (1.5)	14 (1.5)	0.776
DM, n (%)		75 (6.9)	5 (4.7)	39 (8.3)	66 (6.9)	0.669
Hemiplegia, n (%)		0 (0.0)	0 (0.0)	0 (0.0)	0 (0.0)	NA
CKD, n (%)		12 (1.2)	1 (1.6)	4 (1.0)	12 (1.3)	0.944
Solid tumor, n (%)		85 (7.8)	15 (12.5)	50 (10.6)	95 (9.9)	0.125
Leukemia, n (%)		1 (0.2)	1 (0.0)	0 (0.2)	5 (0.6)	0.286
Lymphoma, n (%)		3 (0.4)	1 (0.0)	2 (0.6)	6 (0.7)	0.563
AIDS, n (%)		0 (0.0)	0 (0.0)	0 (0.0)	0 (0.0)	NA
CAD, n (%)		66 (6.1)	3 (3.1)	38 (8.1)	61 (6.4)	0.191
GERD, n (%)		219 (20.1)	21 (17.2)	100 (21.0)	196 (20.4)	0.814
COVID-19, n (%)		1 (0.0)	0 (0.8)	9 (2.1)	64 (6.7)	<0.001
HIV, n (%)		2 (0.3)	1 (0.0)	1 (0.4)	4 (0.5)	0.721
HL, n (%)		351 (32.0)	41 (32.0)	166 (34.7)	317 (33.0)	0.767
Obesity, n (%)	BMI <30	810 (73.9)	95 (74.2)	336 (69.9)	667 (69.1)	0.047
	30 ≦ BMI ≦40	274 (25.0)	31 (24.2)	138 (28.7)	272 (28.2)	
	BMI >40	12 (1.1)	2 (1.6)	7 (1.5)	26 (2.7)	
Osteoporosis, n (%)		65 (6.0)	6 (5.5)	24 (5.2)	44 (4.7)	0.594
Parkinson’s disease, n (%)		2 (0.3)	1 (0.8)	4 (0.8)	5 (0.6)	0.47
RA, n (%)		18 (1.6)	0 (0.0)	9 (1.9)	11 (1.1)	0.326
OSAS, n (%)		116 (10.6)	15 (11.7)	57 (11.9)	118 (12.2)	0.689
Anxiety, n (%)		210 (19.2)	21 (16.4)	95 (19.8)	191 (19.8)	0.824
Depression, n (%)		131 (12.0)	15 (11.7)	57 (11.9)	118 (12.2)	0.995
Arthritis, n (%)		169 (15.4)	15 (11.7)	92 (19.1)	162 (16.8)	0.137
Asthma, n (%)		92 (8.4)	9 (7.0)	30 (6.2)	44 (4.6)	0.006
Autoimmune disease, n (%)		17 (1.6)	2 (1.6)	9 (1.9)	22 (2.3)	0.67
Decreased hearing, n (%)		68 (6.2)	8 (6.2)	23 (4.8)	43 (4.5)	0.304
Decreased vision, n (%)		232 (21.2)	25 (19.5)	107 (22.2)	131 (13.6)	<0.001
Chronic pain, n (%)		16 (1.5)	0 (0.0)	9 (1.9)	21 (2.2)	0.27
Hypertension, n (%)		319 (29.1)	37 (28.9)	165 (34.3)	276 (28.6)	0.132
Hypothyroidism, n (%)		66 (6.0)	11 (8.6)	27 (5.6)	57 (5.9)	0.646

Table 3 shows surgery-related factors. There were significant differences in EBL as well as operation start and end times among the patient groups.

**Table 3:** Surgery-related factors. *: Bold values indicate significance (p < 0.05). AMS: ambulatory status (discharge within 24 hours), OS: observation stay (discharge from 24 hours to 48 hours), inpatient: discharged after 48 hours. EBL = estimated blood loss

Factor	Groups	Pre-pandemic	Restricted period	Post-restricted 2020	Post-restricted 2021	P-value*
Patients (N)		1,096	128	481	965	
Drain use, n (%)		406 (37.1)	39 (31.2)	162 (33.9)	324 (33.7)	0.263
Dural tear, n (%)		29 (2.7)	3 (3.1)	11 (2.5)	29 (3.1)	0.91
Procedure, n (%)	Discectomy	794 (72.4)	108 (84.4)	367 (76.3)	721 (74.7)	0.102
	Laminectomy	265 (24.2)	19 (14.8)	105 (21.8)	227 (23.5)	
	Excision of cyst	25 (2.3)	0 (0.0)	7 (1.5)	10 (1.0)	
	Foraminotomy	8 (0.7)	1 (0.8)	2 (0.4)	6 (0.6)	
	Others	4 (0.4)	0 (0.0)	0 (0.0)	1 (0.1)	
EBL (mL)	Median (range)	25.00 (0.00, 1,718.00)	25.00 (5.00, 1,400.00)	25.00 (5.00, 600.00)	25.00 (5.00, 1,000.00)	<0.001
Procedure time (minutes)	Median (range)	77.00 (9.00, 268.00)	82.50 (33.00, 249.00)	77.00 (13.00, 301.00)	78.00 (16.00, 427.00)	0.388
Number of operated levels, n (%)	1	918 (83.8)	112 (87.5)	388 (80.7)	810 (83.9)	0.216
	2	178 (16.2)	16 (12.5)	93 (19.3)	155 (16.1)	
Operated level, n (%)	L1-2	10 (0.9)	5 (3.9)	5 (1.0)	12 (1.2)	0.167
	L2-3	66 (6.0)	9 (7.0)	29 (6.0)	62 (6.4)	
	L3-4	200 (18.2)	18 (14.1)	102 (21.2)	175 (18.1)	
	L4-5	497 (45.3)	54 (42.2)	230 (47.8)	456 (47.3)	
	L5-S1	318 (29.0)	42 (32.8)	114 (23.7)	258 (26.7)	
Operation start time, n (%)	Morning (before 12:00 p.m.)	481 (43.9)	82 (64.1)	240 (49.9)	466 (48.3)	<0.001
	Early afternoon (12:00 p.m. to 3:30 p.m.)	295 (26.9)	30 (23.4)	131 (27.2)	297 (30.8)	
	Late afternoon (3:30 p.m. to 5:30 p.m.)	173 (15.8)	15 (11.7)	74 (15.4)	141 (14.6)	
	Night shift (after 5:30 p.m.)	147 (13.4)	1 (0.8)	36 (7.5)	61 (6.3)	
Operation end time n (%)	Morning (before 12:00 p.m.)	398 (36.3)	69 (53.9)	200 (41.6)	379 (39.3)	<0.001
	Early afternoon (12:00 p.m. to 3:30 p.m.)	255 (23.3)	33 (25.8)	114 (23.7)	258 (26.7)	
	Late afternoon (3:30 p.m. to 5:30 p.m.)	164 (15.0)	19 (14.8)	80 (16.6)	178 (18.4)	
	Night shift (after 5:30 p.m.)	279 (25.5)	7 (5.5)	87 (18.1)	150 (15.5)	

**Figure 2:**
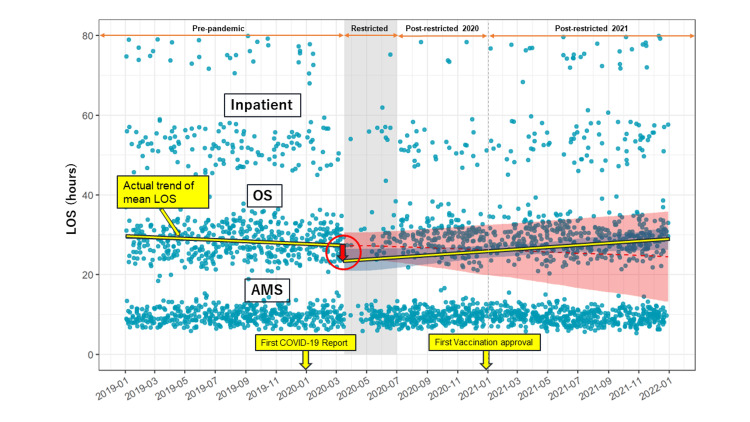
Difference in the length of stay over time. Timeframe difference in LOS among the pre-pandemic, restricted, post-restricted 2020, and post-restricted 2021 periods. Each plot represents a surgical case. AMS: ambulatory surgery patient group (discharged within 24 hours), OS: observation service (discharged from 24 hours to 48 hours), inpatient: discharged after 48 hours. Actual trend of mean LOS (yellow line). The sudden drop in mean length of stay is depicted by the circled red arrow. LOS = length of stay

The retrospective ITS analysis demonstrated a significant drop in mean LOS (coefficient (95% CI) = -9.1 (-16.0, -2.4)) at the beginning of the pandemic during the restricted period that subsequently increased gradually (Figure 2).

**Figure 3:**
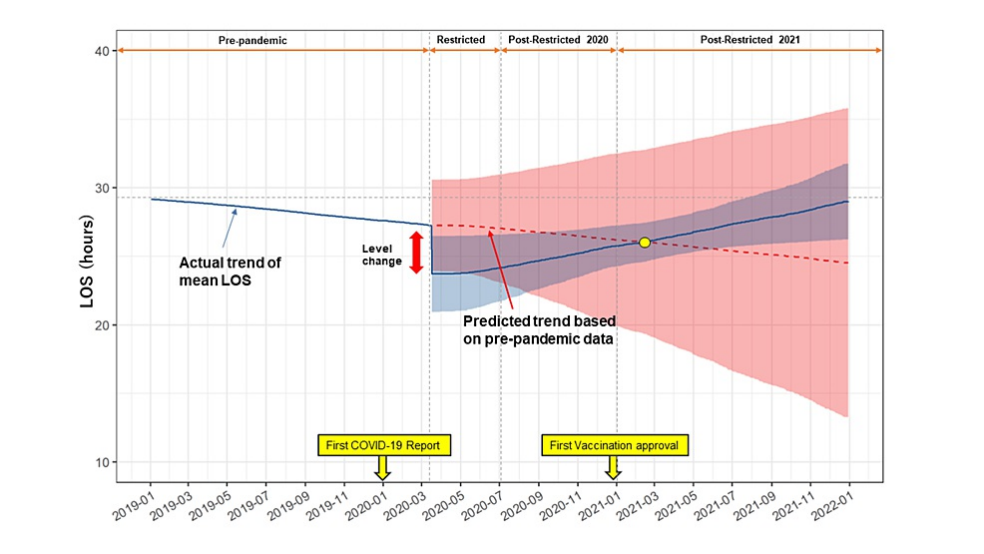
Schematic figure of trend lines. Schematic figure showing trend lines. The colored band represents 95% confidence intervals. The blue line indicates the actual trend of mean LOS. The red dotted line indicates the regressed predicted trend based on pre-pandemic data. LOS = length of stay

The mean LOS gradually increased after the sudden drop at the beginning of the restricted period. The LOS increased and was the same as the pre-pandemic LOS after one year. The regression line showing the actual trend for mean LOS indicated that the LOS gradually recovered by 2021 (Figure 3).

**Figure 4:**
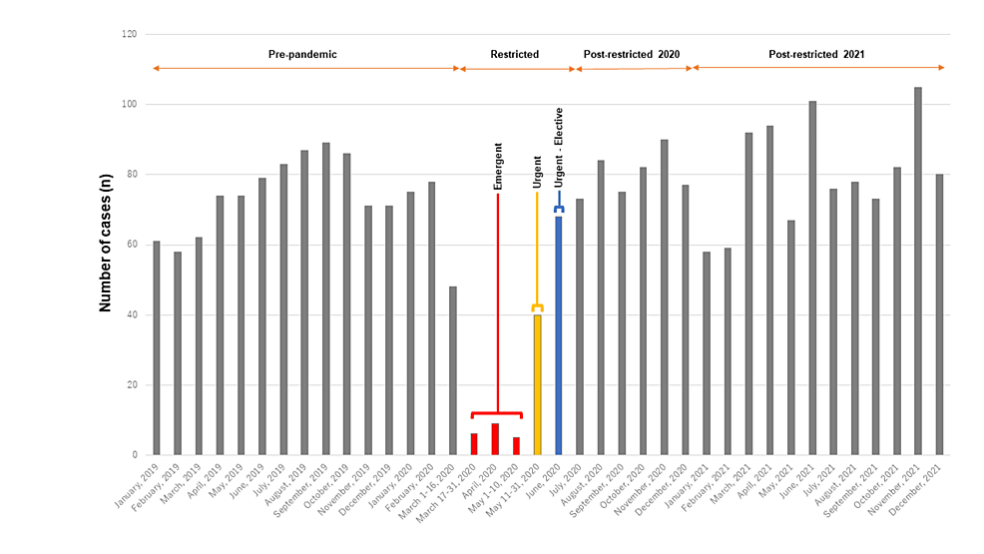
Surgery numbers from 2019 through 2021. Numbers of one to two lumbar decompression AMS cases per month from 2019 through 2021. Gray: pre-pandemic, post-pandemic 2020, and post-pandemic 2021 periods. AMS = ambulatory surgery

In the “Emergent” phase, 20 patients with severe progressing pain and neurological deficits were indicated for surgery, with the oldest patient in their eighties. Of the 20 patients, four showed foot drop. During the “Urgent” phase, restrictions loosened, and the surgery case volume increased to 40 cases. In the “Urgent-Elective” phase, 68 surgeries were performed. Finally, from July 2020 onward, restrictions were completely lifted and all elective surgeries normalized [13] (Figure 4).

Regarding multivariable logistic regression analysis, after adjusting for patient factors, despite the similarities in patient comorbidity and age, multivariate analysis showed that post-restricted 2020 patients still showed a significant association with decreased LOS (Tables 4, 5).

**Table 4:** The results of multivariate analysis. Bold values indicate significance (p < 0.05). CCI = Charlson Comorbidity Index; ASA = American Society of Anesthesiologists physical status; OSAS = obstructive sleep apnea

Factor	Estimate	Standard error	t value	P-value
(Intercept)	-9.05 (-14.73 to -3.37)	2.9	-3.12	0.0018
Age	0.24 (0.12 to 0.35)	0.06	3.94	<0.001
ASA score 2	1.64 (-1.26 to 4.55)	1.48	1.11	0.27
ASA score >3	10.42 (5.72 to 15.11)	2.39	4.35	<0.001
CCI score 1	-2.67 (-5.89 to 0.54)	1.64	-1.63	0.1
CCI score 2 or over	-1.35 (-5.3 to 2.61)	2.02	-0.67	0.5
Drain use	6.40 (4.26 to 8.54)	1.09	5.86	<0.001
Dural tear	34.63 (28.89 to 40.37)	2.93	11.83	<0.001
Estimated blood loss	0.06 (0.04 to 0.07)	0.01	8.1	<0.001
Gender, female	5.12 (3.11 to 7.13)	1.03	5	<0.001
Gender, unspecified	-4.78 (-19.44 to 9.87)	7.47	-0.64	0.52
Laminectomy	6.27 (3.83 to 8.7)	1.24	5.05	<0.001
Maximum pain score	0.93 (0.62 to 1.23)	0.16	5.95	<0.001
Medicare Medicaid	1.18 (-1.75 to 4.1)	1.49	0.79	0.43
Number of operated levels	4.42 (1.72 to 7.11)	1.37	3.21	<0.001
Operation start time (early noon 12:00-15:30)	2.75 (0.51 to 4.99)	1.14	2.4	<0.001
Operation start time (late noon 15:30-17:30)	6.70 (3.91 to 9.48)	1.42	4.72	<0.001
Operation start time (after 17:30)	8.83 (5.38 to 12.27)	1.76	5.02	<0.001
OSAS	4.01 (0.95 to 7.07)	1.56	2.57	<0.001
Surgery duration (minutes)	0.07 (0.04 to 0.09)	0.01	5.02	<0.001
Timeframe restricted period	-2.52 (-7.07 to 2.03)	2.32	-1.08	0.28
Timeframe post-restricted 2020	-2.70 (-5.36 to -0.04)	1.36	-1.99	0.046
Timeframe post-restricted 2021	0.03 (-2.13 to 2.2)	1.1	0.03	0.98
Upper-level surgery	-0.95 (-4.64 to 2.75)	1.88	-0.5	0.62

**Table 5:** Univariate and multivariate analysis for each period. *: Bold values indicate significance (p < 0.05). CI = confidential interval

	Pre-pandemic	Restricted period	Post-restricted 2020	Post-restricted 2021
Univariate analysis				
Unadjusted coefficient (95% CI)	Ref.	-5.72 (-10.86 to -0.58)	-3.15 (-6.16 to -0.14)	-0.70 (-3.13 to 1.73)
P-value		0.029	0.04	0.57
Multivariate analysis				
Adjusted coefficient (95% CI)	Ref.	-2.52 (-7.07 to 2.03)	-2.70 (-5.36 to -0.04)	0.03 (-2.13 to 2.2)
P-value*		0.28	0.046	0.98

## Discussion

Our study demonstrated how the COVID-19 pandemic impacted ambulatory spine surgery at a single orthopedic specialized hospital. During the restricted period, patients were younger (mean = 51.2 years old) and healthier (lower CCI score) with a shorter LOS. Hospital LOS decreased significantly at the beginning of the restricted period then gradually increased and reached pre-pandemic levels within one year. The post-restricted 2020 period was an independent factor affecting the mean LOS.

During the restricted period, our institution continued to function as a specialty musculoskeletal treatment center, but from March 17, 2020, through June 30, 2020, all elective surgeries were restricted with the CMS announcement [5]. Elective surgeries were postponed, delayed, or canceled to conserve resources for COVID-19 patients and protect surgical teams from exposure [5]. Translating the CMS recommendations into practical management and triaging of patients for surgical versus non-surgical treatment presented significant clinical and ethical challenges for spine surgeons [5].

A study with a large number of spine decompression surgery patients in 2019 reported that the mean age was 51.3 years old in New York State [14]. Compared to this study, the mean age at our institution during the pre-pandemic period was older (mean = 56 years old). Patient age decreased during the restricted period to 52 years old and was similar to the mean age of patients who underwent lumbar decompression surgery in New York State in 2019. The reason for the mean age difference is not entirely clear and warrants further investigation. However, as our hospital is an academic orthopedic surgery specialty hospital for elective spine surgery and not emergency trauma, patients may be older for this reason. Lumbar decompression surgeries could result from trauma from younger patients and these patients may go to other hospital trauma centers.

During the pandemic, the patients primarily at risk for death when infected with COVID-19 were the elderly, immunocompromised, and had chronic medical comorbidities, such as heart conditions, chronic kidney disease, liver disease, chronic lung disease, diabetes, or obesity [13]. Surgeries for patients with these risk factors were likely postponed or canceled to avoid the risk of perioperative medical complications that require intensive medical care. This might have led to surgeries on younger and healthier patients during the pandemic.

The sudden drop in LOS at the beginning of the restricted period may be for several reasons. First, as patients were younger and healthier, a shorter LOS may have resulted from an uncomplicated postoperative course. Second, due to a decrease in surgical case volume, most surgeries were completed in the morning before noon. Planned early operation start and end times might have decreased the number of unintended overnight stays and the overall LOS. Third, anecdotally, spine surgeons noted that patients asked to be discharged earlier due to fear of contracting COVID-19 while hospitalized. During the pandemic, the governors of all 50 states in the United States declared states of emergency. To stop the spread of the virus, nearly all state governors issued stay-at-home orders that advised or required residents to shelter in place as the safest place was considered to be one’s home [15].

After the restricted period, the average LOS gradually increased and recovered to pre-pandemic levels in one year. Similarly, patient demographics reverted to what they were originally before the pandemic. Interestingly, after adjusting for patient factors, despite the similarities in patient comorbidities and age, the post-restricted 2020 period was associated with a shorter LOS and was an independent factor for shorter LOS. Since the beginning of the COVID-19 pandemic, there have been a large number of published studies on treatment practices during the pandemic. When searching the keywords “ambulatory surgery” and “spine surgery,” there are nearly 200 publications from 2020 to 2021. Results commenting on patient LOS might be independently influenced by the pandemic. Our study’s findings should be taken into account when interpreting LOS information in these other studies during the post-restricted 2020 period.

Several study limitations exist. First, our analysis did not include any patient-reported descriptions of their pandemic-related concerns due to the retrospective nature of this study. Second, diagnosis, complications, and procedure information were derived from medical insurance billing codes that do not always match the clinical diagnosis. Most importantly, because this study was conducted at an academic orthopedic surgery specialty hospital in an urban area of New York and local COVID-19 situations and public health policies can differ, the generalizability of results may be limited and care must be taken when applying these study results to other practice settings.

## Conclusions

Our results suggest that spine surgery patients from the restricted pandemic period were younger and healthier, which led to a shorter LOS. Hospital LOS significantly decreased at the beginning of the restricted period then gradually increased and recovered to pre-pandemic levels in one year.

As the post-restricted 2020 period itself might be independently influenced by the pandemic, these results should be taken into account when interpreting the LOS of patients undergoing ambulatory lumbar spine surgery in post-restricted phases in the United States.
